# Nanoparticle mediated delivery and small molecule triggered activation of proteins in the nucleus

**DOI:** 10.1080/19491034.2018.1523665

**Published:** 2018-09-14

**Authors:** Hsin-Yi Chiu, Jack A. Bates, Jonas Helma, Hanna Engelke, Hartmann Harz, Thomas Bein, Heinrich Leonhardt

**Affiliations:** a Department of Chemistry and Center for NanoScience (CeNS), Ludwig-Maximilians-Universität München (LMU), Munich, Germany;; b Department of Biology II and Center for NanoScience (CeNS), Ludwig-Maximilians-Universität München (LMU), Planegg-Martinsried, Germany

**Keywords:** Biomolecular complementation, biosensor, controlled release, mesoporous silica nanoparticles, protein delivery, drug induciblity, small molecule control, split venus, nuclear proteins

## Abstract

Protein transfection is a versatile tool to study or manipulate cellular processes and also shows great therapeutic potential. However, the repertoire of cost effective techniques for efficient and minimally cytotoxic delivery remains limited. Mesoporous silica nanoparticles (MSNs) are multifunctional nanocarriers for cellular delivery of a wide range of molecules, they are simple and economical to synthesize and have shown great promise for protein delivery. In this work we present a general strategy to optimize the delivery of active protein to the nucleus. We generated a bimolecular Venus based optical sensor that exclusively detects active and bioavailable protein for the performance of multi-parameter optimization of protein delivery. In conjunction with cell viability tests we maximized MSN protein delivery and biocompatibility and achieved highly efficient protein transfection rates of 80%. Using the sensor to measure live-cell protein delivery kinetics, we observed heterogeneous timings within cell populations which could have a confounding effect on function studies. To address this problem we fused a split or dimerization dependent protein of interest to chemically induced dimerization (CID) components, permitting control over its activity following cellular delivery. Using the split Venus protein we directly show that addition of a small molecule dimerizer causes synchronous activation of the delivered protein across the entire cell population. This combination of cellular delivery and triggered activation provides a defined starting point for functional studies and could be applied to other protein transfection methods.

## Introduction

The cellular delivery of proteins is both a useful method to study cell pathways and a promising possibility for future medicine [1–4]. However, the toolkit for cost-effective and highly functional *in vitro* transfection remains limited, whilst challenges such as short protein serum half-life, inefficient cellular uptake and endosomal entrapment hinder delivery i*n vivo*. Thus, advancements in protein delivery would be useful for cell research and are required to remove a major barrier to the implementation of potential therapies.

Mesoporous silica nanoparticles (MSNs) are a versatile nanocarrier format that has been used for the delivery of chemotherapeutic agents[5,6], oligonucleotides[7,8], and proteins[9–11]. MSNs feature a stable framework to load and shelter cargo from proteases and the immune system and combine high cellular uptake efficiency with flexible surface functionalisation. Diverse surface conjugations permit augmentation of the nanoparticles to enable the modulation of serum half life, selective cell targeting and controlled drug release[12–17]. MSNs can be generated in large volumes at low financial and labor expenditure [18]. Having previously demonstrated the delivery of bioactive proteins using our large pore MSN variant [19] we sought to produce an optimized delivery protocol that maximizes efficiency whilst retaining good biocompatibility, i.e. the integrity and viability of cells. Beside cellular uptake efficiency, the general applicability of MSNs requires that delivered proteins escape the endosomes and retain their function[20].

Several techniques have been developed to quantify efficiencies of cellular protein delivery. The methods often exploit pre-existing cytoplasm to endosome differences such as pH[21], enzyme content[22], access to DNA[23], redox status[24–26] or localization[27] and some utilize the physical separation of the compartments to create exploitable distinctions between the two environments[28,29]. However, despite the advantages of each of these methods, many of these techniques do not provide a real-time readout, give a non-linear output due to amplification, transcription or recombination, give no indication of post-endosome protein functionality, rely on endpoint assays on entire batches of cells, or require complex image analysis. Quantifying delivery efficiencies of functional proteins in real-time still remains challenging. Recently, GFP-based bimolecular fluorescence complementation (BiFC) assays have been developed, consisting of two proteins derived from genetic splitting of a fluorescent protein that only fluoresce after complementation. BiFC assays have been successfully employed for the comparison of cell penetrating peptide (CPP) function and the assessment of antibody internalization[30–32]. They deliver a relative fluorescent readout which is ideal for optimization processes.

In this work, we develop a Venus based optical sensor and apply it to investigate and optimize *in vitro* MSN mediated bioactive protein delivery. We demonstrate how the sensor can be easily applied in conjunction with cell viability assays to optimize diverse aspects of delivery including; total protein transfection, population transfection percentages, cell-to-cell variation, biocompatibility and live-cell delivery kinetics. We demonstrate that large cell-to-cell variability can occur in the delivery of bioactive proteins. As functional studies greatly benefit from defined starting points, we aimed to add another level of control to allow for synchronous and tunable activation of proteins upon delivery.

## Materials and methods

### Materials

Tetraethyl orthosilicate (TEOS, Aldrich, ≥99%), (3-glycidyloxypropyl) trimethoxysilane (GPTMS, Fluka, ≥97%), cetyltrimethylammonium p-toluenesulfonate (CTATos, Sigma), triethanolamine (TEA, Aldrich, 98%), magnesium sulfate anhydrous (99.9%, Sigma), toluene anhydrous (Sigma), bi-distilled water obtained from a Millipore system (Milli-Q Academic A10). N(alpha),N(alpha)-bis(carboxymethyl)-L-lysine hydrate (NTA-lysine, Aldrich), sodium carbonate (Sigma), sodium bicarbonate (Sigma), nickel chloride hexahydrate (Riedel-de Haen), tris(hydroxymethyl)-aminomethane (TRIS, ≥99%, ROTH), acetic acid (99% – 100%, ROTH), thiazolyl blue tetrazolium bromide (MTT, ≥97.5%, Sigma), dimethyl sulfoxide molecular (DMSO, Applichem, biology grade), Dulbecco’s Modified Eagle’s Medium (DMEM, Sigma), Dulbecco’s Phosphate Buffered Saline (PBS, Sigma), FBS Superior (Biochrom, S0615), Gentamycin solution (SERVA, 50 mg/ml), trypsin-EDTA solution (Sigma, T3924), Dulbecco’s Modified Eagle’s Medium – phenol red free (DMEM, Gibco), ethanol (EtOH, Aldrich, absolute).

### Synthesis and functionalization of large pore MSNs

Un-functionalized MSNs were synthesized using a modification of a previously reported procedure [33]. In brief, a mixture of TEA (0.49 g, 3.3 mmol), CTATos (2.73 g, 6 mmol) and H_2_O (144 g, 8 mol) was vigorously stirred (1250 rpm) at 80°C in a 250 ml glass flask until the solution became homogeneous. TEOS (20.83 g, 0.1 mol) was then added and the solution was continuously stirred (1250 rpm) at 80°C for another 2 h, afterwards the synthesized particles can be observed as whitening of the solution. The as-synthesized particles were collected by centrifugation (7000 × g, 15 min) and subsequently subjected to organic template extraction. The organic template extraction was carried out by heating particles in an ethanolic solution (150 mL) containing 3 g of ammonium nitrate at 90 °C under reflux for 1 h followed by a second reflux at 90 °C in a 2 M HCl/ethanolic solution (150 mL) for 1 h. The un-functionalized mesoporous silica nanoparticles (un-MSNs) were collected by centrifugation (7000 × g, 20 min) and were washed with water and EtOH after each extraction step.

To attach epoxy groups to the surface of the MSNs, a post-synthetic grafting procedure was performed. 500 mg of un-MSNs were de-hydrated under reflux (130 °C) in 150 mL of toluene in the presence of MgSO_4_ for 4 h. GPTMS (190 mg, 0.83 mmol, 10 mol% of total silica) was subsequently added to the toluene solution, and the solution was stirred (500 rpm) at 130 °C for 2 h. After the solution had cooled to room temperature, the toluene was removed by rotary evaporator (77 mbar, 45 °C, 250 rpm). The resulting epoxy group-modified MSNs (MSN-Epoxy) were washed three times with 150 mL of EtOH and preserved in 50 mL of absolute EtOH. Centrifugation (7000 × g, 20 min) was used to collect particles after each washing step.

MSN-Epoxy particles were then modified to yield NTA-functionalized MSNs (MSN-NTA). 360 mg of MSN-Epoxy and 200 mg (0.6 mmol) of NTA-lysine were mixed in 10 ml of carbonate-bicarbonate buffer (100 mM, pH 9) and the mixture was stirred at RT overnight. The functionalized MSN-NTA particles were washed three times with 100 mL of tris-acetate (TA) buffer (pH 8) at RT and re-suspended in 36 mL of EtOH. Centrifugation (7000 × g, 20 min) was used to collect particles after each washing step. To immobilize Ni^2+^ on the surface of MSN-NTAs, 5 mg of MSN-NTA was dispersed in 5 ml of NiCl_2_ (50 mM in H_2_O) and stirred at RT for 4 h. The un-bound Ni^2+^ was washed out with H_2_O (three times with 5 mL) and the particles were collected following centrifugation (17000 × g, 5 min). The final NTA-Ni complex modified MSNs (MSN-Ni) were stored in 5 mL of EtOH (MSN concentration: 1 mg/mL) for further use.

### Characterization of MSNs

Scanning electron microscopy (SEM) and scanning-transmission electron microscopy (STEM) were performed at 30 kV on a Helios NanoLab G3 UC instrument (FEI, USA) with a detection system containing a TLD detector and a STEM ADF detector. A drop of EtOH diluted MSN suspension was dried on a carbon-coated copper grid at room temperature for several hours before SEM/STEM observation. Dynamic light scattering (DLS) measurements were performed on a Malvern Zetasizer-Nano instrument equipped with a 4 mW He-Ne laser (633 nm). Nitrogen sorption analysis was performed on a Quantachrome Instrument NOVA 4000e at 77 K. Samples (15 – 20 mg) were degassed at 120 °C under vacuum (10 mTorr) one day before measurement. Pore size distribution curves were obtained based on non-local density functional theory (NLDFT) procedures provided by Quantachrome, using the adsorption branch of N_2_ on silica.

### Plasmid construction and deposition

**Figure 1.**
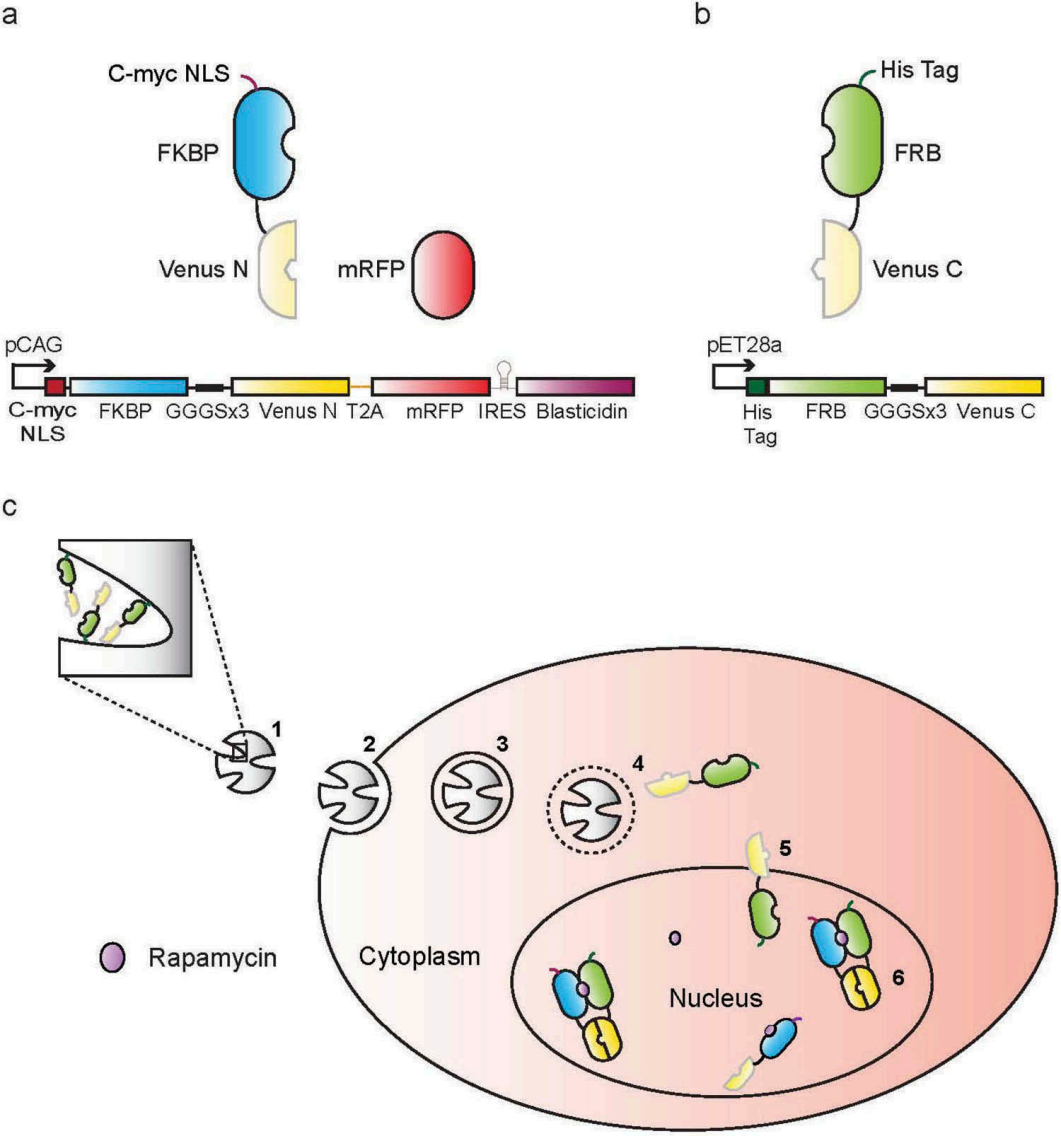
A two-component protein delivery sensor. Layouts of the: **a** mammalian expression cassette and **b** bacterial expression cassette are shown. **c** MSN-mediated protein delivery sensing. **1** His-tagged FRB-VC proteins are loaded into MSNs via surface Ni-NTA complexes. **2** Charged MSN-FRB-VCs bind to HeLa-FKBP-VN cell surfaces and are **3** endocytosed. Lower pH in the endosomal system causes accelerated FRB-VC dissociation from the MSN. **4** Chloroquine shock triggers endosomal protein release followed by **5** free diffusion of FRB-VCs into the nucleus. **6** Addition of rapamycin leads to the formation of FRB/rapamycin/FKBP ternary complexes driving Venus complementation and fluorophore maturation.

A previously described pCAG mammalian expression vector containing an IRES-Blasticidin selection gene following the ORF was used for creation of the pCAG-FKBP-VN-T2A-mRFP cassette[34] (Figure 1a). The protein expression vector pET28a was used for FRB-VC-Histag expression (Figure 1b). The Gibson assembly method was applied for all cloning [35]. Plasmids have been deposited into the addgene repository (pCAG-FKBP-VN-T2A-mRFP: #100979, pET28a-FRB-VC-Histag: #100980).

### FRB-VC protein purification

FRB-VC was expressed in *E. coli* (BL21 strain) and purified via affinity chromatography on a His-trap column. Expression was induced through addition of 0.5 mM of isopropyl beta-D-1-thiogalactopyranoside (IPTG, ROTH) and cells were further cultured at 18 °C overnight. Cells were harvested and lysed in PBS buffer containing 100 μg/ml of lysozyme (Serva, Germany), 2 mM of phenylmethanesulfonyl fluoride (PMSF, sigma) and 25 μg/ml of DNAse (Applichem, Germany) followed by sonication (Branson® Sonifier; 16 × 8 sec, 20% amplitude). Cell debris was collected by centrifugation at 20000 × g for 30 min. Protein purification was performed using a manufacturers 8 M Urea purification protocol (Amersham Biosciences[36]) including renaturation of the protein via FPLC (Äkta Purifier Amersham Biosciences, GE Healthcare, USA) on a 1 ml His-trap column (GE Healthcare, USA). Elution was performed using an increasing imidazole gradient rather than the step-wise imidazole increase outlined in the protocol. Eluted protein was desalted using the PD-10 (GE Healthcare, USA) column and concentrated with an Amicon filter column (cut-off 10 kDa, Merck Millipore, Germany). Purified protein in PBS was aliquoted followed by shock-freezing and storage at −80 °C.

### FRB-VC protein loading to MSN-Ni

1 mg of MSN-Ni was mixed with 500 µg of FRB-VC protein in 500 µl of PBS at 4 °C with shaking (400 rpm) for 1.5 h. The resulting MSN-FRB-VC complexes were collected by centrifugation (3000 × g, 3 min), washed with PBS (1 mL per wash) twice, and re-suspended in 100 µl of PBS.

### Cell culture and stable cell line

HeLa Kyoto cells[37] (HeLa k, a modified HeLa cell line characterized by little cell motility and thus suitable for live cell time-lapse imaging) were cultured in DMEM medium supplemented with 10% FBS and gentamycin (50 µg/ml in cell culture medium) under 5% CO_2_ at 37°C. To generate a cell line stably expressing the pCAG-FKBP-VN-T2A-mRFP construct, pCAG-FKBP-VN-T2A-mRFP was transfected into HeLa k cells using Lipofectamine 3000 reagent (Invitrogen). Blasticidin (10 µg/ml in cell culture medium) was used to select cells between 48 h after transfection and 3 weeks. Highly mRFP fluorescent cells were then isolated via flow cytometry (FACS Aria II, BD Biosciences) to produce a monoclonal cell line (HeLa-FKBP-VN).

### Intracellular protein delivery for MSN concentration optimization

HeLa-FKBP-VN cells in DMEM culture medium were seeded on either a 2-well ibiTreat slide (ibidi, Germany) or a 6-well plate (Corning, USA) at 50% confluency 12 h before the intracellular protein delivery experiment. MSNs loaded with FRB-VC proteins (MSN-FRB-VCs) in PBS were added to cell culture in a serum free DMEM and incubated with cells at 37 °C for 2 h. Afterwards, the residual particles in the medium were washed out using PBS (1.5 mL per well) followed by a short chloroquine shock (0.5 mM in standard DMEM, 1.5 mL per well in cell culture medium, RT, 5 min) to trigger endosomal protein release. Cells were then incubated in fresh cell culture medium (phenol red free). All the assays (live cell imaging, FACS analysis and fluorescence readout) were performed at 24 h post MSN-FRB-VCs addition. Images were acquired on an UltraVIEW VoX spinning dizc system with laser line combiner (PerkinElmer) assembled with an inverted microscope (Axio Observer D1) using a 63x/1.4 NA Plan-Apochromat oil immersion objective (Zeiss). Images were acquired with an EMCCD camera (C9100-50, Hamamatsu). The microscope was equipped with a humidified and heated environmental chamber set to 37 °C, 5% CO_2_ (PeCon). Image acquisition was controlled by the program Volocity (ver. 6.3, PerkinElmer).

### Flow cytometry

Cells were washed with PBS, detached from a 6-well plate using 0.25% Trypsin-EDTA and finally re-suspended in PBS (3 mL per well) prior to flow cytometry (FACS Aria II, BD Biosciences). Data were analyzed using FlowJo (8.1) software. Non-MSN treated HeLa-FKBP-VN-RFP cells were used to gate out dead cells and aggregates and to calibrate appropriate Venus (using FITC settings) and mRFP gating. Venus +ve and – ve cells were analyzed from the RFP +ve group. 10,000 cells were measured per sample. Experiments were triplicated. Error bars represent standard deviations. P values were obtained through application of two-tailed T-tests with unequal variances.

### Microplate reader for cell fluorescence readout

Cells were detached from a 6-well plate using 0.25% Trypsin-EDTA, harvested and washed with PBS. After centrifugation (150 × g, 5 min), cells were re-suspended in 100 µl of PBS and pipetted into a 96-well microplate (Greiner Bio-One, Germany). Fluorescence was measured using a microplate reader (Infinite® M1000 PRO, TECAN) with 515 nm excitation and 528 nm emission for Venus measurements and 556 nm excitation and 586 nm emission for RFP measurements. Four readings were taken per well. Background fluorescence in the Venus channel was measured using the stable cell line without addition of the complementing protein and subtracted from other readings. Measurements were normalized against the RFP channel to account for variations in cell number. Experiments were performed in six biological repeats. Error bars represent standard deviations. P values were obtained through application of two-tailed T-tests with unequal variances.

### MTT assay

One day prior to MTT assay HeLa Kyoto cells were plated on a 96-well microplate (5 × 10^3^ cells per well) in DMEM and incubated at 37 °C. After removal of culture medium, cells were exposed to 100 µl of MSN-DMEM solution per well (serum free) with various concentrations, while the control group was incubated with 100 µl of serum-free DMEM. Following 2 h incubation, the cells were washed with PBS three times to remove the residual particles. Freshly prepared MTT solution (0.5 mg/ml in DMEM) was added to the cells (100 µl/well) and the cells were incubated at 37 °C for another 4 h. The purple crystals metabolized from healthy cells were then dissolved in 100 µl of DMSO and the absorbance was measured at 570 nm, while the reference absorbance was measured at 655 nm using a microplate reader (Infinite® M1000 PRO, TECAN). Experiments were triplicated. Error bars represent standard deviations. P values were obtained through application of two-tailed T-tests with unequal variances.

### Protein delivery rate experiments

#### Rapamycin-primed sample

HeLa-FKBP-VN-RFP cells were seeded onto a 2-well ibi-Treat slide 12 h before the experiment. For the rapamycin-primed protein release tracking experiment, 1 µl of rapamycin (from 250 µM stock solution) and 100 µg of MSN-FRB-VC were mixed in 1 mL of serum-free DMEM, and the mixture was added to the cells before the first image was acquired. After 2 h, particles were washed away using PBS, followed by a chloroquine shock (0.5 mM of chloroquine in standard DMEM, 1.5 mL/well) at RT for 5 min triggering endosomal protein release. Cells were tracked at indicated time points for 24 h. Microscopic images were acquired with a Nikon TiE microscope equipped with perfect focus, Yokogawa CSU-W1 spinning disk unit (50 µm pinhole size), Andor ALC600 laser-beamcombiner: 405nm/488nm/561nm/640nm, Yokogawa CSU-W1 dichroic mirror 405/488/561/640 LD Quad, Andor Borealis illumination unit and Andor IXON 888 Ultra EMCCD camera using a Nikon CFI P-Apo 100x Lambda oil immersion objective NA 1.45. The setup was equipped with an environmental chamber (Okolab BIO 1, Bold Line CO_2_ and temperature module, gas chamber and humidifying module) and controlled by software from Nikon (NIS elements, version 4.51.01). The environmental conditions during the experiment were set to 37 °C, 5 % CO_2_ and humidified atmosphere. Focus drifts during the long-term experiments were compensated by the Nikon perfect focus system. Tiled images (10x10 image fields, 15 % overlap, stitched by NIS elements) were acquired throughout this study to investigate many cells per experiment. 4 color tiles (488 nm, 561 nm, 640 nm and differential interference contrast) were acquired with a frequency of 3 images per hour. On chip binning (2x2) was used throughout to reduce the data amount and to improve the signal to noise ratio. Fluorescence images were acquired with an exposure time of 1 s and an EM gain setting of 160. For the population protein delivery tracking analysis, a single large field area with 200 −300 cells was assessed at each interval.

#### Rapamycin-delayed sample

For the Venus reconstitution kinetics tracking (rapamycin-delayed sample), 100 µg of MSN-FRB-VC were mixed in 1 mL of serum-free DMEM, and the mixture was added to the cells before the first image was acquired. After 2 h, particles were washed away using PBS, followed by a chloroquine shock. Cells were tracked at indicated time points using confocal microscopy (an UltraVIEW Vox spinning dizc confocal system, PerkinElmer, UK). Rapamycin (1 µl, final conc. 250 nM in standard DMEM) was added to the sample 22 h after chloroquine shock. Live cell images were acquired at randomly chosen areas to prevent Venus fluorescence bleaching. 200 – 300 cells were imaged and counted at each time point. Venus positive cells were counted visually. Venus positive cells in % correspond to the number of nuclei with Venus fluorescence divided by the number of cells with RFP fluorescence * 100.

### Single cell fluorescence tracking

Tracking of fluorescent intensity within single cells was performed on images acquired using a Nikon TiE microscope utilized as outlined above. Rapamycin-primed (250 nM rapamycin applied with MSNs) and delayed experiments (250 nM rapamycin added 24 hs after MSNs) were performed. Fiji software was used to analyze images. A circular ROI of consistent size was used to measure fluorescence in cell nuclei. The ROI was positioned in the nucleoplasm excluding the darker nucleoli. Measurements were taken until mitosis of the cell occurred or imaging ended.

## Results

To develop a controllable BiFC sensor we used the chemically inducible dimerization (CID) components FKBP/FRB[38] that form a heterodimer in the presence of rapamycin[39]. For BiFC we selected the split Venus fluorescent protein (split 154/155) based on its high fluorescence signal, rapid maturation at 37 °C and low auto-complementation[40,41]. Our overall approach is outlined in Figure 1. In brief, we fused the N-terminal portion of Venus (VN) with FKBP and added a c-Myc nuclear localization signal (NLS)[42] to facilitate subsequent image analyses (Figure 1(a)). For expression control and calibration we added an mRFP which follows a T2A peptide[43]. The T2A induces ribosomal skipping which enables the synthesis of two proteins (FKBP-VN and mRFP) from one transcript. A blasticidin resistance gene was included to assist in the selection and maintenance of a monoclonal stable cell line (HeLa-FKBP-VN), which was used for all subsequent studies. The complementary half of the BiFC/CID complex (Figure 1(b)), consisting of a His-tagged FRB domain fused to the C-terminal half of Venus (VC), was bacterially expressed, purified by affinity chromatography and loaded into the MSNs for cellular delivery (Figure 1(c)).

Following endocytosis of FRB-VC loaded MSNs (MSN-FRB-VC)[44], carrier and cargo proceed through the endosomal system where decreasing pH causes dissociation of His-tagged FRB-VC from the MSN. Endosomal rupture is triggered using chloroquine[19] and FRB-VC is released to the cytosol. FRB-VC diffuses to the nucleus and interacts with FKBP-VN, which is primed for dimerization with pre-bound rapamycin. This ternary complex formation (FKBP-rapamycin-FRB) approximates the two Venus halves, promotes complementation and enables chromophore maturation. Thus, successful protein delivery can then be monitored as Venus fluorescence in the nucleus, which concentrates the signal, facilitates automated image analysis and improves the signal to noise ratio.

**Figure 2.**
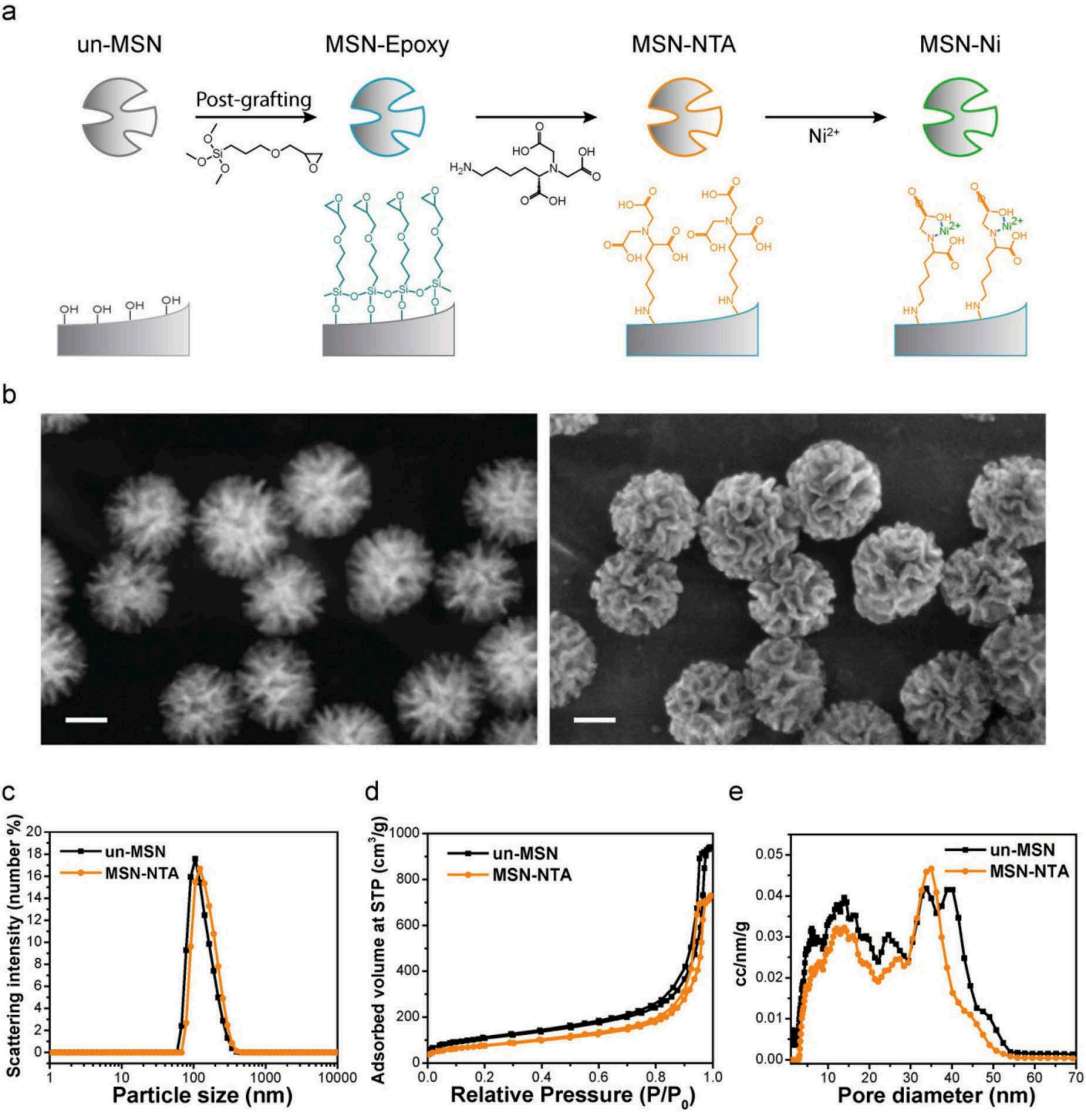
Synthesis and characterization of MSNs for controlled binding and release of His-tagged protein. **a** Surface modification series of un-functionalized MSNs (un-MSNs) to MSN-Ni. **b** STEM (left) and SEM (right) images of MSN-NTAs, *scale bar: 50 nm*. Characterization of un-MSNs and MSN-NTAs: **c** dynamic light scattering, **d** N_2_ sorption isotherms and **e** pore size distribution calculated via NLDFT mode.

Large-pore MSNs were surface-functionalized with NTA-Ni complexes (MSN-Ni) through a series of modifications (Figure 2(a)) to accommodate pH dependent binding and release of the His-tagged FRB-VC[10,11,19]. STEM and SEM images (Figure 2(b)) indicated that the resulting nitrilotriacetic acid-modified MSNs (MSN-NTAs) exhibit uniform particle size and coral surface morphology with irregular pore shape (pore size ranging from 10 – 40 nm) consistent with the structure of the un-functionalized form (Figure S1). These particle characteristics were confirmed by dynamic light scattering (DLS) (Figure 2(c)) and N_2_ sorption analyses (Figures 2(d,e)) and are summarized in Table S1.

**Figure 3.**
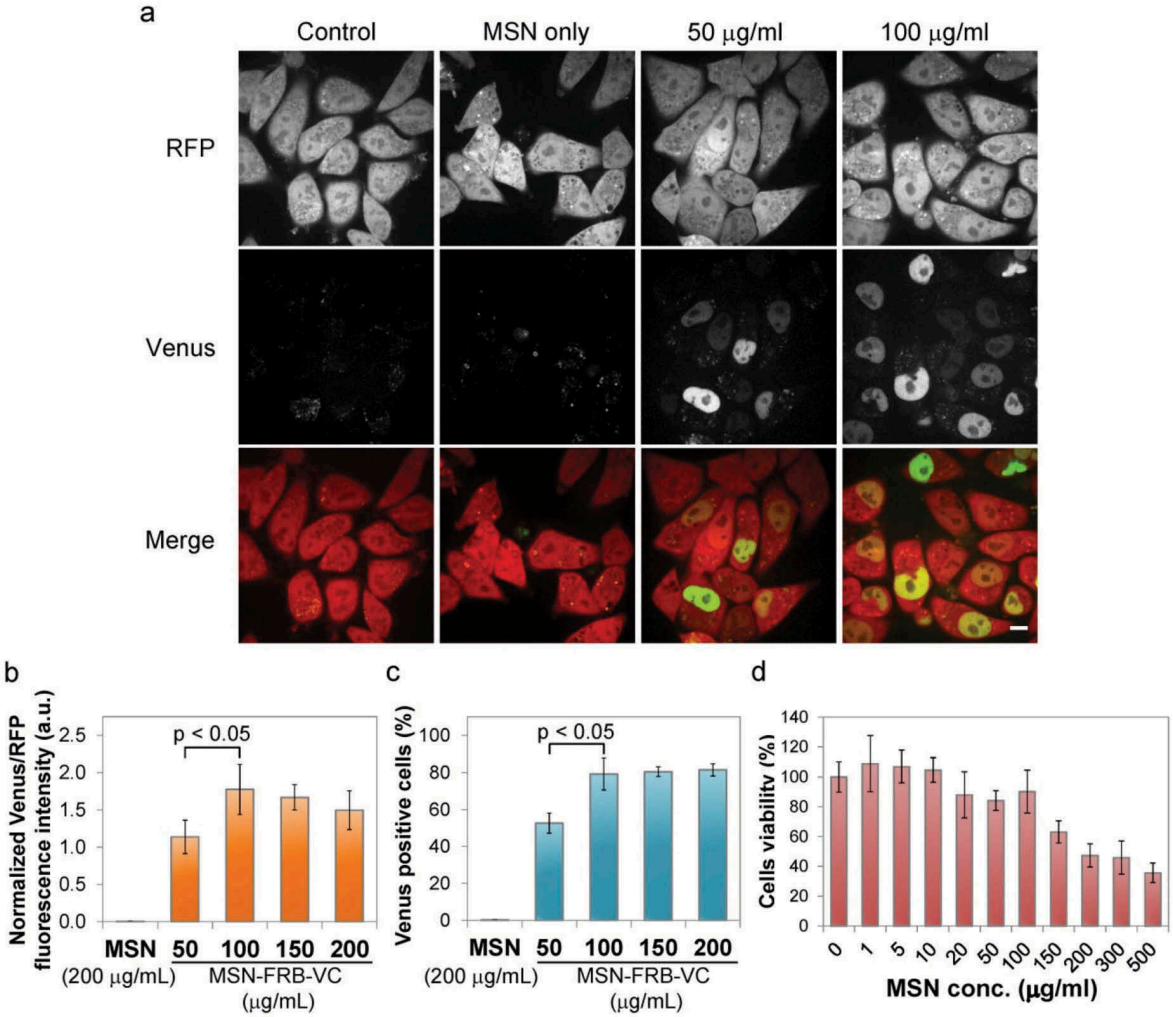
Optimization of MSN-mediated protein delivery. Different MSN-FRB-VC concentrations were incubated with HeLa-FKBP-VN cells. **a** Live-cell images were taken 20 h post endosomal protein release induction. *Scale bar: 10 µm*. **b** Population fluorescence was measured using a microplate reader. **c** Flow cytometry measured protein transfection rate. **d** MTT assays assessed cytotoxicity of un-MSNs. Error bars represent SDs.

To optimize protein delivery we first tested different particle concentrations. As expected, the HeLa-FKBP-VN cell line by itself and the addition of unloaded MSNs did not yield any Venus fluorescence while MSN-FRB-VC complexes led to clear nuclear signals (Figure 3(a), Figure S2). Successful protein delivery can be conveniently quantified in cell populations with microplate fluorometry, as the formation of Venus fluorescence requires endosomal escape and release of functional proteins. Whilst a significant increase (p = 0.004) in protein delivery is seen between the 50 µg/ml and 100 µg/ml concentrations, the fluorescent signal plateaued at higher concentrations (Figure 3(b)). We then measured protein transfection rates, i.e. the fraction of cells with a clear fluorescence signal, by flow cytometry and again found a significant increase from 50 µg/ml to 100 µg/ml concentrations (p = 0.016) indicating some dose dependency, with higher concentrations having no further positive effect (Figure 3(c)). MTT assays were performed in parallel to assess possible cytotoxicity. Results indicated that nanoparticle additions up to 100 µg/ml did not affect cell viability. However, increased cytotoxicity was observed with higher particle concentrations of 150 µg/ml and 200 µg/ml (Figure 3(d)). Based on these three data sets, we selected 100 µg/ml as the optimal MSN concentration to obtain high protein delivery efficiency with low cytotoxicity.

**Figure 4.**
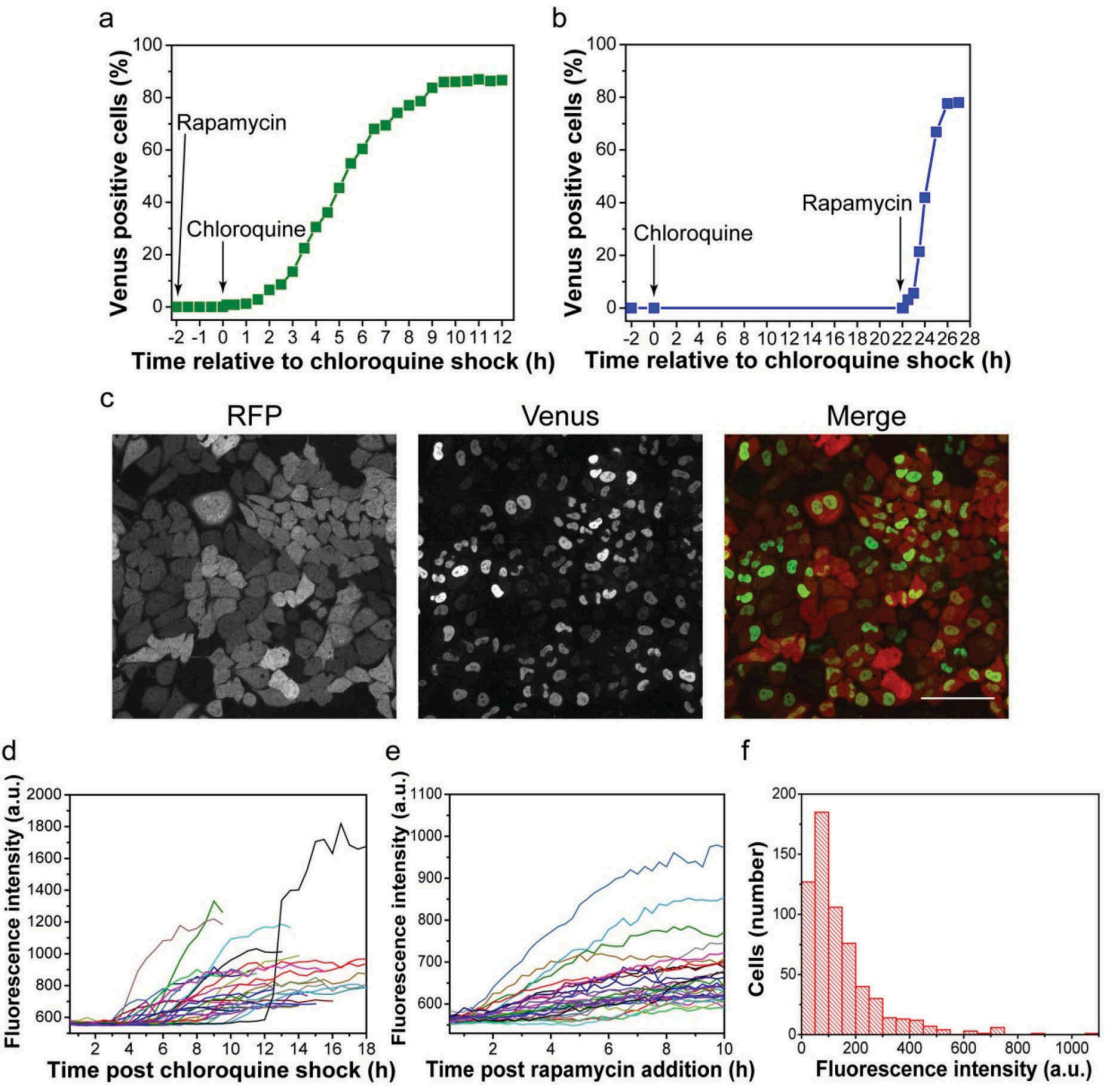
Live cell tracking of protein release. **a** MSN-FRB-VCs were added to rapamycin primed HeLa-FKBP-VN cells at time point −2 h and live cell imaging began. Following chloroquine shock at 0 h cells were imaged and analyzed for Venus nuclear fluorescence rates for a further 12 h. **b** MSN-FRB-VCs and chloroquine were applied as above. Delayed rapamycin addition (22 h after chloroquine shock) triggered synchronous activation of delivered protein, cell imaging and analysis of Venus fluorescence continued for 5 h. **c** A live cell image of the rapamycin-primed sample 10 h after chloroquine shock. *Scale bar: 100 µm*. Fluorescence intensity tracking from 30 cells in **d** rapamycin-primed and **e** rapamycin-delayed samples. **f** Fluorescence intensity distribution among 625 cells in a rapamycin-primed sample analyzed at 15 h post chloroquine shock.

To monitor protein delivery in single cells, we imaged live cell populations with spinning dizc microscopy over time. We first incubated rapamycin primed HeLa-FKBP-VN cells with MSN-FRB-VC complexes (100 µg/ml) and imaged before and after chloroquine shock. While no Venus fluorescence was detected before the chloroquine shock, first signals became visible in nuclei of about 1% of the cells within 30 min after chloroquine mediated endosome rupture, surpassed 50% after 5.5 h and reached 85% at 9 h (Figure 4(a)). A live cell image of protein release taken 10 h post chloroquine shock shows yellow fluorescent nuclei, i.e. successful protein delivery, in most cells (Figure 4(c)). Time-lapse videos covering the entire time course of protein delivery into hundreds of cells show this cell-to-cell temporal variability of nuclear fluorescence onset over 24 h (movie 1).

To address this cell-to-cell temporal variability we incubated cells with MSN-FRB-VC and added rapamycin (250 nM) 22 h after chloroquine shock (Figure 4(b)). We reasoned that at this point the protein was likely already released in the majority of the cells and therefore the timing of Venus fluorescence should represent sensor complex formation and maturation rather than protein release. The comparison of both experimental conditions shows that while in rapamycin primed cell populations the onset of nuclear fluorescence occurred over a period of 10 hours, the later addition of rapamycin caused a synchronous onset within 1–2 h within the majority of cells monitored by live cell microscopy (Figures 4(a,b)). Also, the quantification of fluorescence in single cells over time shows the heterogeneity in fluorescence increase in the rapamycin primed cells as opposed to the synchronous increase of fluorescence in the cells with delayed rapamycin addition (Figures 4(d,e)). The heterogeneous fluorescence onset and increase in the rapamycin primed cells suggests that endosomal rupture may continuously occur even several hours after transient chloroquine shock. Consistently, we observe enlarged endosomes even 15 h after chloroquine shock indicating prolonged endosomal rupture over many hours (Figure S3). Unlike sensors that rely upon transcription or other amplification mechanisms, the ratio-metric nature of the BiFC sensor enables cell-to-cell variation measurements not only between but also within populations (Figure 4(f)). With image analysis, parameters such as the range of protein released within a cell population as well as means and standard deviations can be determined. Clearly, the combination of protein delivery with subsequent activation methods, like the bimolecular complementation triggered here by rapamycin, provides a defined starting point for functional studies.

## Discussion

The BiFC sensor described in this study makes it possible to simultaneously monitor the time course of protein delivery and cellular responses in thousands of cells over hours and days. The lack of any signal amplification mechanism limits the maximal sensitivity of the BiFC sensor, but due to its ratio-metric nature enables cell-to-cell variation measurements. In consideration of the significance of cellular protein stoichiometry this should be a useful attribute for understanding and adapting nanocarriers for various tasks.

Fluorescent live cell delivery tracking revealed a variable timing of bioavailability upon addition of MSNs, indicating that cellular uptake and intracellular release of functional proteins varies from cell to cell by several hours. This temporal heterogeneity in direct protein delivery experiments complicates functional studies. One possible solution is protein splitting and addition of controllable dimerization modules that allows for delivery of inactive protein halves over longer time intervals and subsequent activation by chemical induction across cell populations. Our results show that the delivery of inactive protein halves in combination with the chemically inducible dimerization components FKBP/FRB allow for rapamycin triggered functional complementation and thus provide a defined starting point for functional studies and enable the controlled manipulation of cellular functions.

The focus of this study was on the delivery process and therefore only one half of the split protein was delivered and the other half was produced by genetic means in the recipient cells, but as single MSNs can carry multiple molecules and single cells can uptake multiple MSNs, also mixtures of proteins can be delivered. To be able to quantify the rate and efficiency of delivery we used a split fluorescent protein (Venus) which is only one of a rapidly growing list of proteins that can be split and then functionally complemented. Therefore, this approach could easily be extended to a large number of proteins through either trial and error or based on structural information and should be applicable to single globular domains as well as multidomain proteins [45,46].

## Conclusions

In summary, we have developed an inducible two-component fluorescent live cell sensor to track and optimize protein delivery with respect to efficiency, bioavailability and biocompatibility. We propose that the system described here is well suited for multi-parameter optimization and the comparison of different protein nanocarriers. We demonstrate that large pore MSN-Nis are capable of non-toxic nuclear delivery in up to 80% of a cell population whilst requiring only a his-tag for protein loading. In light of these properties as well as the prevalent use of his-tags for protein purification we suggest that MSN-Nis are good candidates for widespread cell research use. Finally, we have shown that small molecule controlled bimolecular complementation can be used for synchronous activation of delivered proteins in cell populations and provide a defined start point for functional studies.

## Availability of data and materials

All data generated or analyzed during this study are included in this published article and the Additional information files.

Plasmids for the production of sensor proteins and cell lines are available through addgene repository (pCAG-FKBP-VN-T2A-mRFP: #100979, pET28a-FRB-VC-Histag: #100980).
