# Supplementary material for: Nanoparticle mediated delivery and small molecule triggered activation of proteins in the nucleus
Source: Nucleus. 2018 Sep 14;9(1):530–42. doi: 10.1080/19491034.2018.1523665 (PMC6244737; doi:10.1080/19491034.2018.1523665)
Supplement: Supplemental Material [file kncl-09-01-1523665-s001.docx]

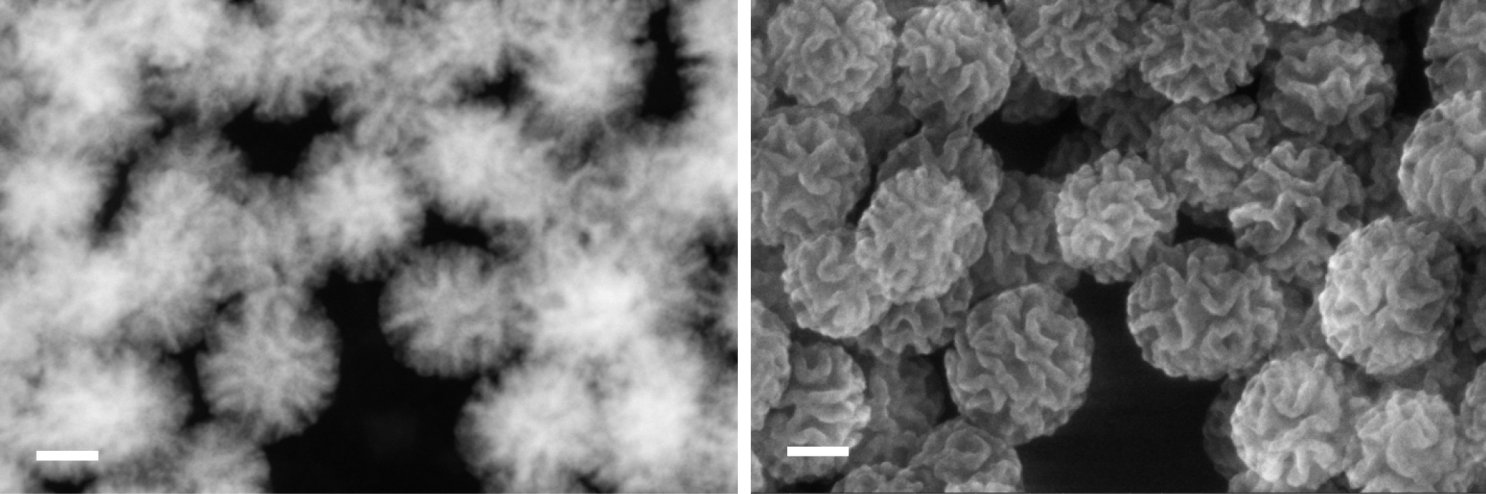


Figure S1. STEM (left) and SEM (right) images of un-functionalized MSNs. *Scale bar: 50 nm.*

| Sample | Particle size (nm) | Pore size distribution (nm) | Pore volume (cm^3^/g) | Surface area (m^2^/g) |
| --- | --- | --- | --- | --- |
| Un-MSN | 133 ± 50 | 4 - 45 | 1.4 | 390 |
| MSN-NTA | 152 ± 54 | 4 - 40 | 1.1 | 275 |

Table S1. Summary of characterization data of un-MSNs and MSN-NTAs.


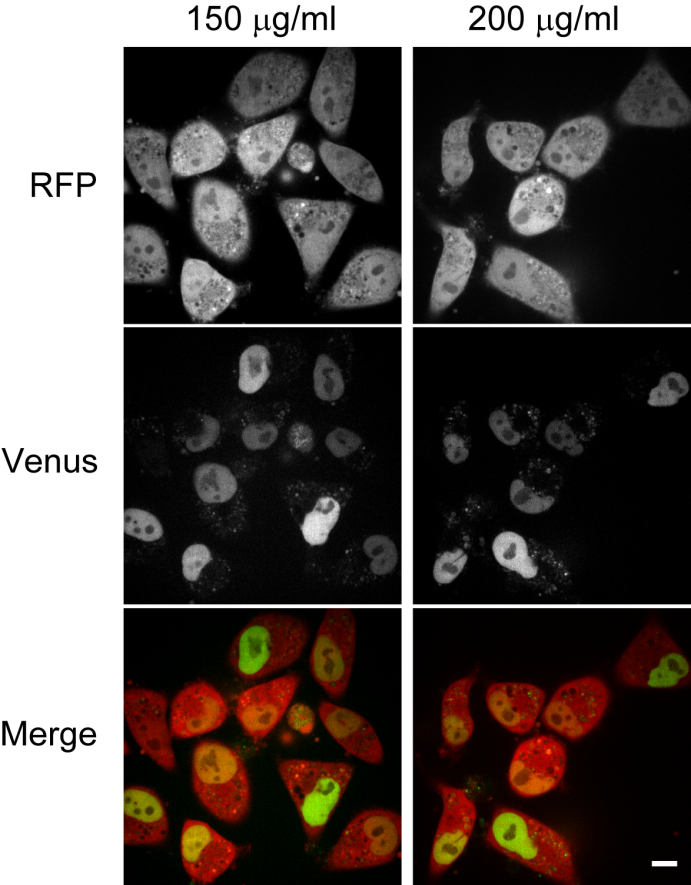


Figure. S2 Live cell confocal imaging of MSN-mediated intracellular protein delivery in the cytosolic protein delivery detection system. HeLa-FKBP-VN cells were incubated with 150 µg/ml and 200 µg/ml of MSN-FRB-VC, respectively, and images were taken 20 h post endosomal release trigger (chloroquine shock). *Scale bar: 10 µm.*


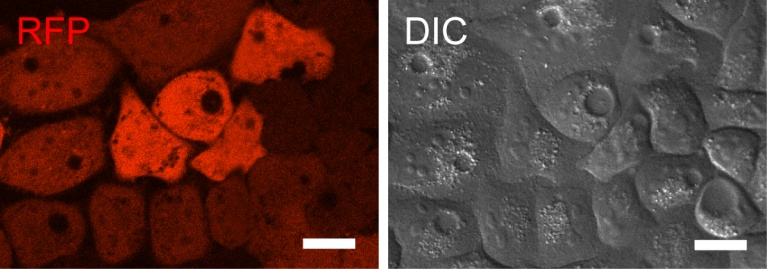


Figure. S3 Live cell images at 15 h post chloroquine shock. Swollen vesicles can be observed in cells after 15 h of chloroquine shock. *Scale bar: 10 µm.*
